# Targeting COL6A3-C5 with nigericin suppresses endotrophin formation and enhances insulin sensitivity in obesity

**DOI:** 10.1038/s12276-026-01661-y

**Published:** 2026-03-05

**Authors:** Chu-Sook Kim, Woobeen Jo, Jungsun Yoo, Min Kim, JIn-Pyo An, Won-Keun Oh, Jiyoung Park

**Affiliations:** 1 Department of Biological Sciences, College of Information and Biotechnology, Ulsan National Institute of Science and Technology, Ulsan, Republic of Korea; 2 Research Institute of Pharmaceutical Sciences, College of Pharmacy, Seoul National University, Seoul, Republic of Korea; 3 Graduate School of Health Science and Technology, College of Information and Biotechnology, Ulsan National Institute of Science and Technology, Ulsan, Republic of Korea

**Keywords:** Metabolic disorders, Type 2 diabetes, High-throughput screening

## Abstract

Endotrophin, a cleavage product of collagen VIα3 (COL6A3), contributes to fibroinflammation in adipose tissue and exacerbates systemic insulin resistance in obesity. Previously, we demonstrated that various hypoxia-induced matrix metalloproteinases (MMPs) are directly involved in the cleavage of COL6A3 to generate endotrophin in obese adipose tissue; thus, inhibition of endotrophin generation by blocking MMP access could be beneficial for treating obesity-related metabolic disease. Here we identified nigericin as an inhibitor of endotrophin generation, which improves fibroinflammation and insulin sensitivity in both hypoxic adipocytes in vitro and diet-induced obese mice in vivo. Mechanistically, nigericin directly binds to the COL6A3-C5 domain, competing with MMPs and thereby disrupting the interactions between the COL6A3-C5 domain and MMPs. This interference prevents the cleavage of endotrophin from the COL6A3 by MMPs, ultimately inhibiting its generation. Taken together, these results strongly suggest that pharmacological blockade of endotrophin cleavage, by using nigericin, effectively decreases endotrophin levels and improves endotrophin-mediated fibroinflammation and insulin resistance in obesity. Furthermore, this new therapeutic strategy could be applied to various metabolic diseases and solid tumors where endotrophin levels are pathologically elevated.

## Nigericin reduces endotrophin and improves insulin sensitivity

Obesity can lead to health problems due to changes in fat tissue, including fibrosis. This study focuses on a protein called endotrophin (ETP), which is linked to these changes and related diseases such as diabetes and cancer. Researchers explored how a natural compound called nigericin might help. The study involved feeding mice a high-fat diet to induce obesity and then treating them with nigericin. The researchers measured the effects on ETP levels and insulin sensitivity. They found that nigericin reduced ETP production by interfering with enzymes called MMPs that help produce ETP. This reduction improved insulin sensitivity in the mice. Results suggest that nigericin could be a potential treatment for obesity-related conditions by targeting ETP. The study highlights the importance of finding new ways to manage obesity and its complications.

This summary was initially drafted using artificial intelligence, then revised and fact-checked by the author.

## Introduction

Adipose tissue (AT) fibrosis is a critical pathological feature of obesity that actively drives metabolic deterioration. Hypoxic stress and chronic inflammation within expanding adipose depots promote excessive deposition and remodeling of extracellular matrix (ECM) components, thereby impairing adipocyte function and disrupting systemic metabolic homeostasis. Among the various ECM components involved, type 6 collagen (COL6) plays a particularly prominent role in this process. COL6 constitutes a major portion of the ECM within AT^1^. Increased levels of COL6 and its cleavage product, endotrophin (ETP), in obese AT have been implicated in obesity-related metabolic diseases^2–4^. ETP exerts profibrotic, proinflammatory and angiogenic effects in the microenvironment of various metabolic organs and cancers, playing a crucial role in the pathogenesis of diverse metabolic conditions, such as obesity-related type II diabetes mellitus, chronic liver disease and various cancers^2,5–9^. Elevated ETP levels in obese AT has been linked to diabetes development^2^. Furthermore, among patients having type 2 diabetes, elevated ETP levels is a risk marker for mortality, as well as kidney and cardiovascular complications^10^. Serum ETP has also been investigated as a prognostic biomarker for chronic fibrotic disease progression^11–14^, and classifying and/or monitoring patients with metabolic derangements^15–20^. Therefore, ETP may be an important therapeutic target for the treatment of obesity-related metabolic diseases and cancers.

Neutralization of ETP activity through therapeutic monoclonal antibodies reverses AT fibroinflammation, improving insulin sensitivity in high-fat diet (HFD)-fed obese mice^2^, and suppresses tumor growth and enhances chemosensitivity in both polyomavirus middle T antigen (MMTV-PyMT) mouse models and xenografts of human breast cancer cells in vivo^5,6,8^. Moreover, pharmacological agents, such as RAS and SGLT2 inhibitors, indirectly reduce ETP levels by inhibiting fibroinflammatory markers, such as TNFR-1, IL-6 and MMP7, thereby reducing ECM turnover in fibrogenesis and alleviating chronic profibrotic responses in diabetic nephropathy^21,22^. Furthermore, rosiglitazone, along with other PPARγ agonists, indirectly lowers ETP levels by suppressing COL6 expression, leading to reduced AT fibroinflammation and improved insulin sensitivity^23^. These findings highlight the therapeutic potential of targeting ETP, both directly and indirectly, for the treatment of obesity-related metabolic diseases and cancers. However, to the best of our knowledge, no pharmacological agents that directly suppress ETP generation have been identified.

Various proteinase mediates cleavage of COL6A3 to produce ETP. Bone morphogenetic protein 1 (BMP1) and matrix metalloproteinase (MMP)14 have been suggested to cleave COL6A3 by targeting distinct cleavage sites^8,24,25^. We reported that various MMPs, including MMP2, MMP9 and MMP16, directly mediate stepwise cleavage of COL6A3 to efficiently produce ETP^26^. Hypoxia is an upstream regulator of COL6A3 expression and the proteolytic processing that regulates ETP generation. Meanwhile, hypoxia-dependent suppression of miR-29 expression derepress MMP2, MMP9 and MMP16 expression, contributing to increased COL6A3 cleavage and ETP production in hypoxic AT^26^. Indeed, adenovirus-mediated miR-29 overexpression in AT of obese mice reduced adipose ETP production and improved glucose and insulin tolerance in adipocyte-specific HIF-1α-knockout (KO) mice^26^. This suggests that targeting HIF-1α, MMPs and/or miR-29 in combination can provide metabolic benefits by reducing ETP production in obese AT. Based on our current understanding of ETP generation, developing drugs that target both HIF-1α and MMPs axes may be a potential approach for treating obesity-related metabolic disorders. Therefore, the aim of this study was to develop small molecules that simultaneously inhibit both HIF-1α and MMPs within the AT of obese mice to efficiently suppress ETP generation, thereby improving fibroinflammation and systemic insulin sensitivity.

## Materials and methods

### Animals

Mice were housed in cages under a 12-h dark–light cycle, with free access to water and standard diet. For the HFD feeding experiments, mice were fed a diet that contained 60% of calories from fat (D12492, Research Diets) for 7 weeks. After 7 weeks of HFD feeding, mice were randomly divided into four groups and intraperitoneally injected with indicated drugs (Vehicles, 0.4NGC,1.2NGC, Rosi) twice per week for 3 weeks (*n* = 4–5 per group). All experiments were conducted using littermate control male mice at 7 weeks old. All animal protocols were approved by the Ulsan National Institute of Science and Technology Institutional Animal Care and Use Committee (UNISTIACUC-22-53/19-35).

### OGTT and ITT

For the oral glucose tolerance test (OGTT), mice were fasted for 4 h before being administered a glucose dose of 2 g/kg via oral gavage (VWR, cat. no. 50-99-7). For the insulin tolerance test (ITT), the mice also underwent a 4-h fasting period before receiving an intraperitoneal (i.p.) injection of insulin at 0.75 units/kg (HI0210, Humulin R, Lilly). Blood glucose levels were then assessed at specified intervals using a blood glucose meter (Accu-Chek) through tail vein clipping.

### Western blotting

Following cell lysis, total protein underwent electrophoresis on 8% or 15% sodium dodecyl sulfate–polyacrylamide gel electrophoresis (SDS–PAGE) and was subsequently transferred to nitrocellulose membranes. The nitrocellulose membrane was blocked with 5% skim milk, and immunoblotting was conducted using antibodies against HIF-1α, human and mouse ETP, FN, αSMA, p-AKT, AKT, p-JNK, JNK, p-ERK, ERK, β-actin, V5 and FLAG. After primary antibody incubation, membranes were treated with secondary antibodies labeled with an infrared dye emitting at 800 nm (Li-Cor Bioscience). Data analysis was performed using Odyssey software (version 2.1, Li-Cor Bioscience). Supplementary Table 1 provides details regarding the antibodies used in this study.

### Histology

For immunohistochemical investigations, ATs were excised, fixed in phosphate-buffered saline (PBS)-buffered 10% formalin for 1 day, and washed with 50% ethanol and distilled H_2_O. Following paraffin embedding, 4-μm sections underwent deparaffinization. After antigen retrieval with citrate buffer and blocking with 5% bovine serum albumin and 0.3% H_2_O_2_, sections were stained using a primary antibody against ETP, Mac2, a biotinylated secondary antibody (cat. no. 65-6140; Life Technologies) and streptavidin/HRP (1:250; cat. no. P0397; Agilent Dako). Secondary antibodies were detected using the DAB+ Substrate Chromogen System (Agilent Dako) following the manufacturer’s protocol. The slides were counterstained with filtered Harris hematoxylin. Image acquisition was performed using an FSX100 microscope (Olympus). Details about the antibodies used in this study are provided in Supplementary Table 1.

### Immunoprecipitation (IP)

HEK293T cells transiently transfected with the expressing vectors were lysed with IP lysis buffer containing 50 mM Tris–HCl (pH 8.0), 150 mM NaCl and 1% NP-40, along with protease inhibitors, followed by centrifugation at 13,000 rpm for 15 min. After protein quantification, 800 μg of cell lysates were incubated with 20 µl of PBS prewashed Anti-FLAG Affinity gel (cat. no. B23102, Selleckchem) for 2 h at 4 °C on a rotating shaker. Following five rounds of centrifugation (200*g*, 30 s) with IP lysis buffer washing, beads were resuspended in 50 µl of SDS sample loading buffer and boiled at 99 °C for 8 min. For western blotting analysis, 25 µl was loaded.

### Assessment of serum biochemical parameters

Serum triglycerides (AM157S-K), cholesterol (AM 202-K), alanine aminotransferase (ALT) (AM102-K), aspartate aminotransferase (AST) (AM103-K) and blood urea nitrogen (BUN) (AM165-K) were measured in accordance with the manufacturer’s protocol.

### Chemicals and reagents

The chemicals and reagents used in this study are listed in Supplementary Table 2.

### Cell culture

Differentiation of 3T3-L1 cells was conducted according to a previously established protocol^26^. Pre-adipocyte cells were grown in Dulbecco’s modified Eagle medium (DMEM) supplemented with 10% fetal bovine serum (FBS) at 37 °C and 5% CO_2_ until reaching full confluence. Subsequently, the growth medium was replaced with differentiation medium consisting of insulin, dexamethasone and methylisobutyl-xanthine in DMEM with 10% FBS for 48 h at 37 °C and 10% CO_2_. The differentiation medium was refreshed every 48 h for 8 days with adipocyte maintenance medium containing insulin in DMEM with 10% FBS at 37 °C and 10% CO_2_. Differentiated 3T3-L1 cells were cultured under either hypoxic (1% O_2_) or normoxic (21% O_2_) conditions at 37 °C for 24 h.

### RT–qPCR

Reverse-transcription quantitative polymerase chain reaction (RT–qPCR) was performed as previously described^26^. Total RNA were extracted from cells using TRIzol (Favorgen), followed by RT–qPCR performed on an ABI 7500 using the SYBR TOPreal qPCR premix kit (Enzynomics). The 18S rRNA served as the internal control. Data normalization was conducted using the 2^−ΔΔCt^ method for relative quantification. The primer sequences are provided in Supplementary Table 3.

### ETP cleavage biosensor

Construction of the ETP-cleavage biosensor was previously described^26^. ETP cleavage activities of MMPs were assessed using a luciferase assay system (Promega). HEK293T cells were transfected with the ETP cleavage biosensor, MMPs and a β-galactosidase (β-gal) expressing vector using Lipofectamine 2000 (Invitrogen). Small-molecule compounds were then treated and incubated for 24 h, and conditioned media and cell lysates were used for measuring luciferase activity and β-gal activity, respectively. β-Gal activity in cell lysates was used for normalization.

### HRE luciferase assay

Luciferase activity of HIF-1α was measured by using a luciferase reporter vector harboring five copies of the hypoxia-responsive element (HRE) in the promoter and HIF-1α-PPN vector, a DNA construct containing the HIF-1α cDNA with alanine substitutions at Pro402, Pro563 and Asn 803, thereby allowing constitutive HIF-1α expression^27^. HEK293T cells were transiently transfected with a reporter vector, HIF-1α-PPN vector and β-gal-expressing vector with or without drug candidates. Cell lysates were used for analysis of luciferase and β-gal activity. Normalization was performed on the basis of β-gal activity in cell lysates.

### Prediction of protein–protein interactions

The binding complex of ETP and the catalytic domain of MMP9 was predicted using the AlphaFold Server Multimer model based on AlphaFold 3. The predicted complex structure was visualized using PyMOL ver.3.0.5, and residues involved in interactions were identified using the protein interface preset function with a 3.5-Å cutoff. Interaction residues of ETP were substituted with alanine to generate the mutant form. The binding complex of mutant ETP and the catalytic domain of MMP9 was then predicted and visualized following the same procedure.

### Molecular docking analysis

Molecular docking between the C5 domain of COL6A3 and the FN II domain of MMP9 was performed using the CDOCKER and ZDOCK modules implemented in BIOVIA Discovery Studio 4.0 (Accelrys). The predicted C5 domain structure exhibited high confidence (predicted local distance difference test >90), and residues R3122, F3124 and W3141 within the binding pocket were identified as key interaction sites. The C5 domain of COL6A3 and the FN II domain of MMP9 were defined as the receptor and ligand, respectively, with a rotational sampling step of 6°. All structures were prepared by removing water molecules and bound ligands, adding hydrogen atoms, assigning partial charges and performing energy minimization using the Adopted Basis Newton–Raphson algorithm. The top-ranked docking poses were selected on the basis of CDOCKER interaction energy and analyzed for hydrogen bonding, π-alkyl and van der Waals interactions.

### MD simulation

All-atom molecular dynamics (MD) simulations were performed using the CHARMM36 force field. The C5–FN II complex was solvated in an orthorhombic water box with at least 20 Å padding on all sides and neutralized with counterions. Energy minimization was carried out using the SMART Minimizer algorithm. The system was gradually heated to 300 K over 100 ps, followed by a 10-ns production run using nanoscale molecular dynamics (NAMD), with trajectory frames recorded every 10 ps.

### Surface plasmon resonance (SPR) analysis

Protein affinity was assessed using a Biacore X100 instrument (GE Healthcare). Recombinant human ETP and MMP9 protein were generated using transient transfection of vectors expressing ETP and MMP9 proteins, respectively, in HEK293T cells. Following purification using fast protein liquid chromatography, protein integrity was confirmed using SDS–PAGE analysis. After pH scouting of ETP (pH4.5), it was immobilized on a carboxymethylated dextran-coated gold surface of a Biacore Series S Sensor Chip CM5 (GE Healthcare, BR-1005-30). The analyte, MMP9 (10 μM) was flowed across the surface of a sensor chip immobilized with the ligand (ETP) containing nigericin (NGC) at 0–1 μM in a running buffer (HBS-EP buffer; GE Healthcare, BR100188, 0.01 M HEPES pH 7.4, 0.15 M NaCl and 3 mM EDTA). Following injection, dissociation of MMP9 was allowed for 120 s. The regeneration of the ligand was achieved with 20 mM NaOH for 120 s after each analyte injection. Experimental conditions were maintained at a temperature of 25 °C and flow rate of 30 μl/min. Analyses were performed using Biacore X100 evaluation software.

### Statistical analysis

The results are expressed as the mean ± s.e.m., and statistical significance among groups was assessed using either the two-tailed Student’s *t*-test or one-way analysis of variance (ANOVA). *P* < 0.05 was considered significant. Graphs and statistical analyses were created using GraphPad Prism 9 software.

## Results

### Identification of small-molecule inhibitors for suppressing ETP generation

**Fig. 1:**
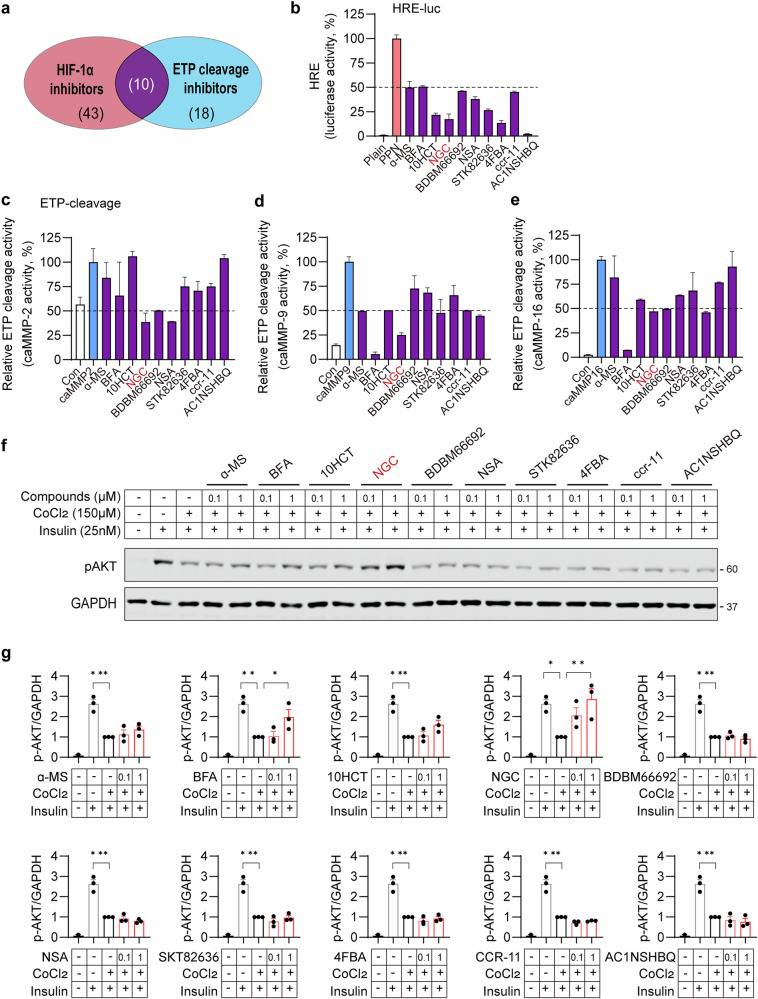
Screening for small-molecule compounds that inhibit ETP production. **a** Schematic overview of the screening strategy to identify compounds capable of repressing both HIF-1α and ETP cleavage activity. **b** HIF-1α inhibitory activity of identified compounds. The HIF-1α activity was assessed by cotransfection of the HIF-1α-PPN expression plasmid with a HRE-luciferase reporter construct into HEK293T cell following incubation with natural compounds. The values were normalized to β-gal activity. **c**–**e**, ETP cleavage activity of identified compounds. Luciferase activities of the ETP-cleavage biosensor were determined using caMMP2 (**c**), caMMP9 (**d**) and caMMP16 (**e**). HEK293T cells were cotransfected with the ETP-cleavage biosensor and each caMMP-expressing vector following incubation with natural compounds, and luciferase activity was measured in the conditioned media. **f** Insulin responsiveness of identified compounds. The 3T3-L1 adipocytes were treated with CoCl_2_ (150 µM) for 48 h, followed by natural compounds for 16 h, before stimulation with insulin (25 nM) for 15 min. The levels of insulin-stimulated AKT phosphorylation were determined using immunoblotting. GAPDH was used as a loading control. **g** Quantification of pAKT levels normalized to GAPDH from the immunoblot analyses shown in panel f. Statistical significance was evaluated using one-way ANOVA. *P < 0.05, **P < 0.01, ***P < 0.005 (*n* = 3 per group). 10HCT, 10-hydroxycamptothecin; α-MS, alpha-mangostin; NSA, necrosulfonamide; 4FBA, 4-fluorobenzaldehyde.

ETP is generated through MMP-mediated cleavage from its precursor, the COL6A3 chain^26^. Effective inhibition of ETP requires coordinated suppression of COL6A3 expression, MMP expression and MMP activity at both transcriptional and post-translational levels. To identify small-molecule inhibitors of ETP, we screened over 1,000 commercially available compounds derived from natural sources, including plants, fungi, soil and the ocean, using a luciferase reporter system containing an HRE promoter, as HIF-1α is a key transcriptional regulator of COL6A3 and MMPs involved in ETP generation^26^. We further screened for inhibitors against ETP cleavage at the protein levels by using ETP-cleavage biosensor^26^ as described in the ‘Materials and methods’. As multiple MMPs contribute to ETP release by targeting its cleavage site, we assessed the activity of an ETP-cleavage biosensor for multiple MMPs, including MMP2, MMP9 and MMP16. Among the tested compounds, 53 compounds inhibited HIF-1α activity by at least 50% at 1 µM in the HIF-1α reporter assay, while 28 compounds reduced ETP cleavage activity by 50% at 1 µM. Through these screenings, ten compounds from both screening systems were identified as candidates (Fig. 1a).

To further validate these compounds, HEK293T cells were cotransfected with the HRE-luciferase reporter gene and HIF-1α-PPN (P402A/P564A/N803A mutant), which allows stable expression of HIF-1α, even in normoxia^27^. The cells were then treated with 1 µM of the ten selected compounds for 24 h, revealing that all compounds effectively inhibited HIF-1α reporter activity (Fig. 1b). HEK293T cells overexpressing the catalytic domains of MMP2, MMP9 and MMP16 were transfected with an ETP-cleavage biosensor and treated with 1 µM of each compound for 24 h. NGC showed the strongest inhibition of MMP2 activity, reducing ETP cleavage by over 50% at 1 µM (Fig. 1c). In addition, seven and four of the ten compounds inhibited MMP9 and MMP16 activity responsible for ETP release, respectively (Fig. 1d,e).

Given that ETP reduces insulin sensitivity in adipocytes during obesity^3,4^, we further evaluated candidate compounds for their effects on CoCl₂-induced insulin resistance by assessing insulin-stimulated Akt activation in 3T3-L1 adipocytes. This analysis identified two compounds, brefeldin A (BFA) and NGC, that effectively restored insulin signaling (Fig. 1f,g). Based on these findings, NGC, the top-ranked candidate, was selected for in-depth investigation of its potential to suppress ETP generation and improve obesity-induced insulin resistance.

### NGC as a novel drug candidate for suppressing ETP generation

**Fig. 2:**
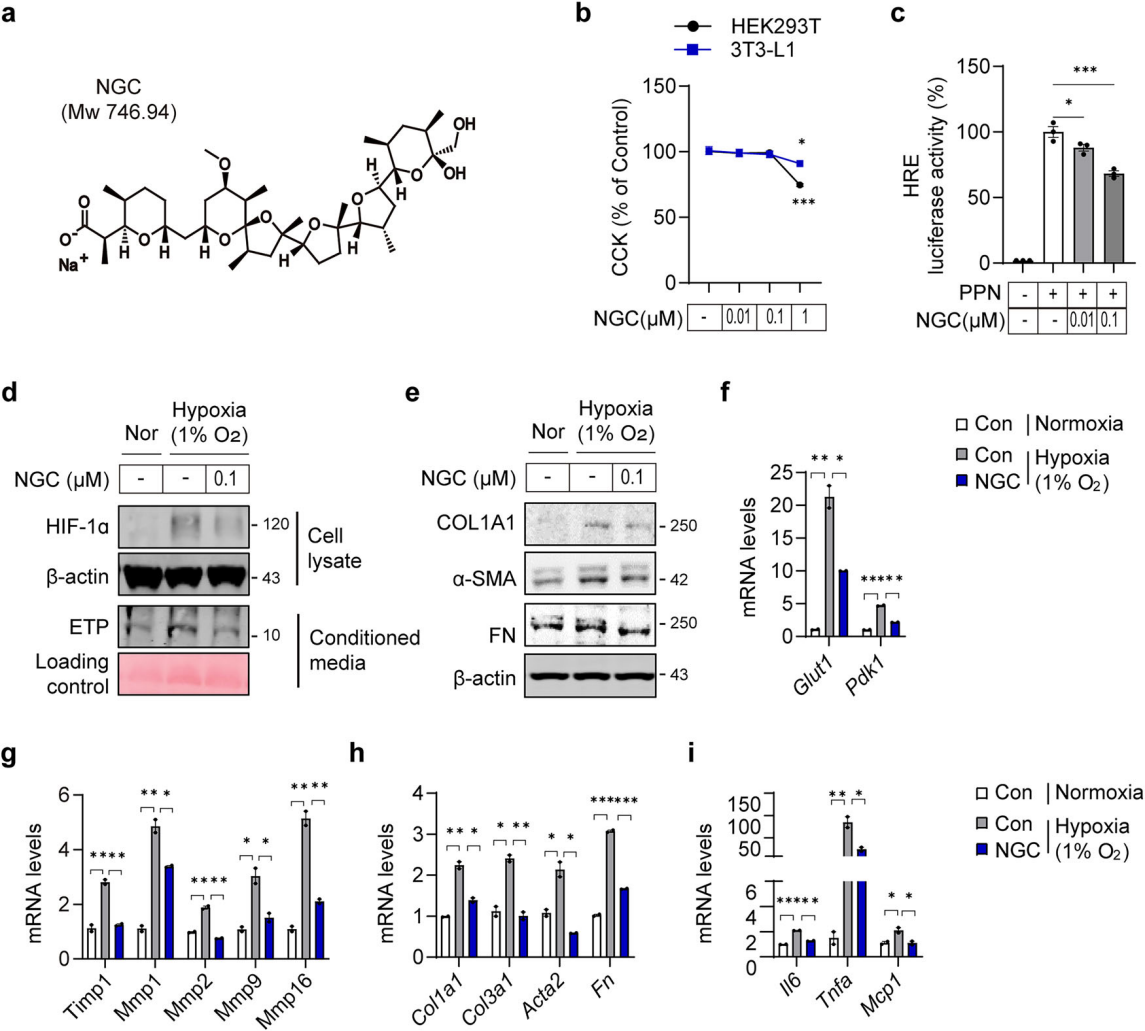
NGC ameliorates HIF-1α expression, ETP production and fibrosis in hypoxic 3T3-L1 adipocytes. **a** Chemical structure of the selected small molecule, NGC. **b** Assessment of NGC cytotoxicity in HEK293T and 3T3-L1 adipocytes with various concentrations (*n* = 5 per group). **c** Luciferase assay of HIF-1α activity was determined in the HEK293T (*n* = 3 per group). **d**, **e** The levels of HIF-1α, secreted ETP (**d**) and profibrotic proteins (**e**) in 3T3-L1 adipocytes were analyzed using immunoblotting following treatment with NGC under hypoxia compared with those under normoxia for 24 h. β-Actin and Ponceau staining were used as loading controls, respectively. **f**–**i** The mRNA levels of HIF-1α target genes (**f**), *MMPs* (**g**) and fibrosis- genes (**h**) and inflammation-associated genes (**i**) in 3T3-L1 adipocytes following treatment with 0.1 µM NGC under hypoxia compared with those under normoxia for 24 h. Statistical significance was evaluated using one-way ANOVA. *P < 0.05, **P < 0.01, ***P < 0.005.

NGC is a monocarboxylic polyether ionophore primarily secreted by *Streptomyces* species (Fig. 2a), exhibiting potent antibacterial, antifungal, antimalarial and anticancer activities^28–31^. NGC selectively transports monovalent cations (Na⁺ and K⁺), disrupting ionic balance and transmembrane potential^32^, and it activates the NLRP3 inflammasome by inducing K⁺ efflux, leading to caspase-1 activation and IL-1β secretion, contributing to its antimicrobial and anticancer effects^33–35^. More recently, NGC has demonstrated protective effects in streptozotocin-induced diabetic pregnancy by reducing hyperglycemia-induced oxidative stress, improving lipid profiles and decreasing embryo lethality, highlighting its therapeutic potential in diabetic pregnancy^36^. However, its metabolic impacts on obesity-induced metabolic disorders remain unclear. So far, no insights are available on how NGC modulates fibroinflammation and insulin sensitivity in AT during obesity.

To assess the cytotoxicity of NGC, we treated NGC to 3T3-L1 adipocytes and HEK293T cells, confirming no toxicity at concentrations below 0.1 µM (Fig. 2b). We then evaluated its effect on ETP generation by measuring hypoxia-induced HIF-1α activity and ETP levels with or without NGC treatment. HIF-1α transcriptional activity, assessed via an HRE-reporter assay, was significantly reduced by NGC in a dose-dependent manner (Fig. 2c). Similarly, HIF-1α protein levels and ETP release in conditioned media were increased in hypoxic conditions (1% O₂, 24 h), both of which were markedly reduced by NGC in 3T3-L1 adipocytes (Fig. 2d). Furthermore, NGC decreased the expression of myofibroblast markers, including COL1A1, α-SMA and fibronectin, under hypoxic conditions (Fig. 2e). This effect was further confirmed by the reduced expression of HIF-1α target genes (Fig. 2f), ECM-modifying enzymes (Fig. 2g), fibrosis (Fig. 2h) and inflammatory marker genes (Fig. 2i) in adipocytes. These findings suggest that NGC mitigates hypoxia-induced fibrosis and inflammation, at least in part, by suppressing ETP generation via HIF-1α inhibition.

To further clarify how NGC suppresses HIF-1α activity, we examined HIF-1α regulation at multiple levels. RT–qPCR analysis showed that NGC does not affect *HIF-1α* mRNA expression (Supplementary Fig. 1a), indicating that transcriptional regulation is not involved. By contrast, cycloheximide-chase assays revealed that NGC accelerates HIF-1α protein degradation (Supplementary Fig. 1b), thereby reducing its stability. Moreover, cellular fractionation demonstrated that NGC markedly decreases the nuclear translocation of HIF-1α (Supplementary Fig. 1c). Collectively, these results indicate that NGC suppresses HIF-1α activity not by regulating its transcription, but by promoting its post-translational destabilization and limiting its nuclear accumulation.

### NGC decreases ETP levels without affecting MMP proteolytic activity

**Fig. 3:**
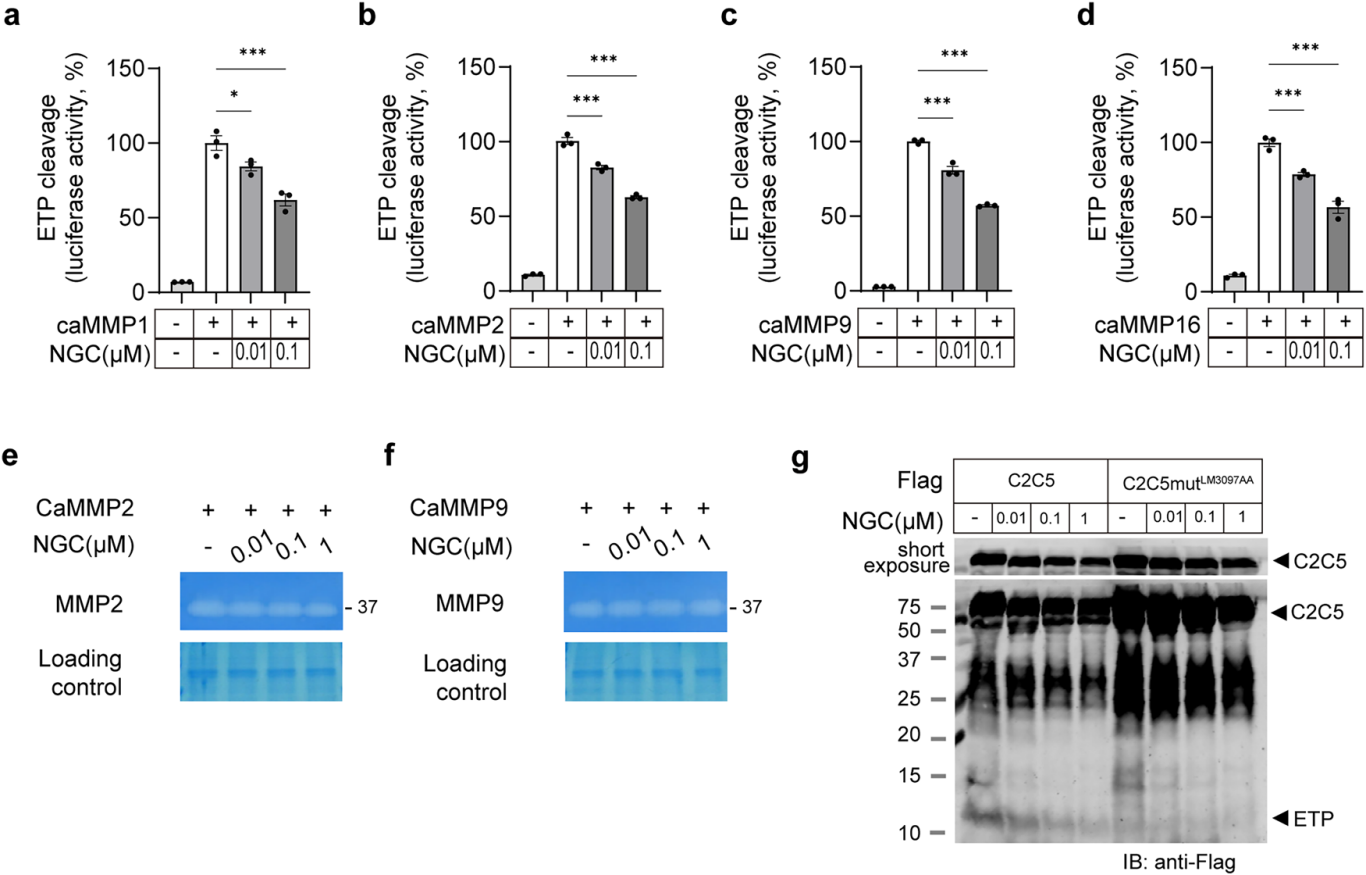
NGC blocks COL6A3 cleavage without affecting MMP catalytic activity. **a**–**d** Luciferase activities of the ETP-23AA cleavage biosensor were determined using multiple catalytic MMPs (caMMP1 (**a**), caMMP2 (**b**), caMMP9 (**c**) and caMMP16 (**d**)). Statistical significance was evaluated using one-way ANOVA. *P < 0.05 (*n* = 3 per group). **e**, **f** Activity of MMPs was measured in conditioned medium using gelatin zymography. Conditioned media of HEK293T cells, transiently transfected with caMMP2 (**e**), or caMMP9 vector (**f**), were incubated with the indicated NGC for 16 h and analyzed using zymography. **g** Validation of NGC specificity on ETP cleavage activity with C2C5–FLAG constructs containing point mutations at cleavage sites (L/M). ETP, cleaved from C2C5 by endogenous MMPs, was detected using immunoblotting with anti-FLAG antibodies and suppressed by NGC. The L/M mutant failed to release ETP regardless of NGC treatment.

Next, we investigated which MMPs are affected by NGC-mediated suppression of ETP generation. An ETP-cleavage biosensor was cotransfected with the catalytic domain of MMP1 (caMMP1), caMMP2, caMMP9 and caMMP16—enzymes known to directly cleave the Col6a3-C5 domain—in HEK293T cells^26^. ETP cleavage activity was then measured with or without NGC treatment. Notably, NGC inhibited all MMP-mediated ETP cleavage (Fig. 3a–d), suggesting it may act as a broad-spectrum MMP inhibitor. To determine whether NGC directly suppresses the proteolytic activity of MMPs, we performed a gelatin zymography assay using conditioned media from either caMMP2- or caMMP9-overexpressing HEK293T cells, treated with or without NGC. Notably, MMP2 and MMP9 are gelatinases that proteolytically degrade gelatin; their gelatin digestion activities appeared as clear bands against a dark-stained background. However, NGC treatment did not alter the proteolytic activity of either caMMP2 or caMMP9 (Fig. 3e, f).

To further confirm that NGC reduces ETP generation by inhibiting its cleavage from COL6A3, we generated a C2C5-LM mutant via site-directed mutagenesis. This mutant contains the COL6A3 C2–C5 domains with mutations at the LM3097AA sites. This mutation disrupts a critical site within the C5 domain of COL6A3 required for ETP release^26^. While ETP was autonomously cleaved from wild-type C2C5 without exogenous MMPs and dose-dependently reduced by NGC, the C2C5-LM mutant failed to release ETP regardless of NGC treatment (Fig. 3g). These findings strongly indicate that NGC reduces ETP levels by preventing its release rather than directly inhibiting MMP proteolytic activity.

### Direct interaction between COL6A3-C5 domain and MMP is required to generate ETP

How does NGC suppress ETP release from the COL6A3 precursor at the protein level without affecting MMP proteolytic activity? Several mechanisms may contribute to MMP activity toward specific substrates, including substrate specificity, activation, localization, inhibition by tissue inhibitors of metalloproteinases (TIMPs), allosteric regulation by ECM components and post-translational modifications^37–39^. Among these, the recognition and binding of MMPs to their target substrates are crucial steps for substrate cleavage^40^. MMPs contain specific substrate recognition domains distinct from their catalytic domains, such as the hemopexin-like domain and fibronectin II (FN II) repeats. MMP2 and MMP9 contain three FN II repeats within their catalytic domains, which enhance substrate binding affinity and proteolytic activity^41,42^. Deletion of the FN II repeats from the MMP9 catalytic domain results in an 80% loss of activity toward an interstitial collagen model triple-helical peptidase substrate^43^. Similarly, MMP2 is activated through its FN II repeats, which bind to collagen; in the absence of this binding, its gelatinase activity is significantly reduced^44,45^. Given the critical role of FN II repeats in substrate binding, NGC may suppress ETP release by disrupting MMP–COL6A3 interactions rather than directly inhibiting MMP proteolytic activity.

**Fig. 4:**
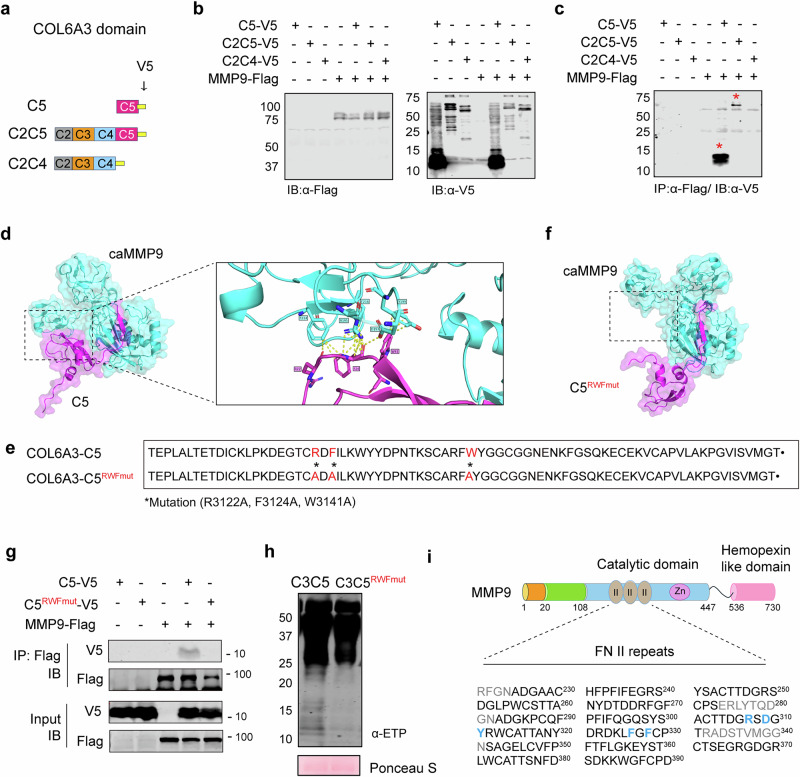
MMPs recognize C5 domain of COL6A3 protein to proteolytically cleave ETP. **a** Schematic illustration of the DNA construct for various domains of COL6A3 used in this experiment. The C2–C5 (C2C5), C2–C4 (C2C4), and C5 domains were tagged with V5 at the carboxyl terminus. **b**, **c** Direct interaction between MMP9 and indicated COL6A3 domains. HEK293T cells were transiently transfected with MMP–FLAG and each COL6A3 domain construct. **b** Cell lysates were immunoblotted using anti-FLAG and anti-V5 antibodies. **c** Immunoblots with anti-V5 antibodies followed by IP with FLAG antibodies. Red asterisks indicate specific binding between MMP9 and COL6A3 domain construct. **d** Left: bioinformatics simulation showing the predicted combination of ETP (magenta) and caMMP9 (cyan) using the AlphaFold predictive algorithm. Right: magnified image presenting the interaction site of COL6A3-C5 with caMMP9. Each interacting protein residue is highlighted in matching-colored sticks and labeled. **e** Red marks represent possible binding amino acids of COL6A3-C5. Charged amino acids substituted with alanine are asterisked in red (COL6A3-C5^RFWmut^; R3122A, F3124A and W3141A). **f** The 3D structure of COL6A3-C5^RFWmut^ (magenta) and caMMP9 (cyan) predicted by alpha-fold predictive algorithm. **g** Direct interaction between COL6A3-C5 and MMP9. HEK293T cells were transiently transfected with indicated MMP9–FLAG, and either the COL6A3-C5 or COL6A3-C5^RFWmut^ construct. Cell lysates were immunoblotted using anti-FLAG and anti-V5 antibodies. Immunoblots for MMP9, COL6A3-C5 and COL6A3-C5^RFWmut^ expression, followed by IP with FLAG antibodies. **h** Generation of ETP cleaved from either the COL6A3-C3C5 (pre-ETP) or COL6A3-C3C5^RFWmut^ construct was analyzed using immunoblotting with anti-ETP antibodies. **i** Schematic diagram of the domain structure of MMP9. The fibronectin type II (FN II) repeat domain, which is critical for substrate binding, is located within the catalytic domain. Black marks indicate the tripartite FN II repeat. Amino acid residues at the predicted binding sites with COL6A3-C5 are highlighted in blue based on AlphaFold predictions.

To investigate whether NGC inhibits the substrate recognition and binding of MMPs for COL6A3, thereby preventing ETP release, we identified the COL6A3 domains that directly interact with caMMP9. MMP9 exhibited the strongest effect on ETP generation among multiple MMPs^26^; therefore, it was selected for further assessments to elucidate the mechanism of action of NGC in suppressing ETP release. We performed co-IP following the cotransfection of FLAG-tagged MMP9 with COL6A3 containing various carboxyl domains, such as COL6A3-C5, COL6A3-C2C5 and COL6A3-C2C4 in HEK293T cells (Fig. 4a). The expression of each construct was confirmed using immunoblotting (Fig. 4b). MMP9 directly interacted with the C5 and C2C5 domains, whereas no interaction was observed with C2C4 (Fig. 4c), suggesting that MMP9 specifically binds to the C5 domain of COL6A3. This domain, also known as the Kunitz-like domain and referred to as ETP, undergoes proteolytic cleavage during COL6 filament maturation^46^; however, unlike other Kunitz domains, it does not exhibit serine protease inhibitory activity^47^.

Using the AlphaFold predictive algorithm, we constructed a three-dimensional (3D) docking model of COL6A3-C5 and caMMP9, highlighting the interacting residues as color-coded stick representations (Fig. 4d). To determine whether the predicted amino acids within the C5 domain are crucial for MMP binding, we generated point mutations, replacing the predicted active binding amino acids with alanine (COL6A3-C5^RFWmut^; R3122A, F3124A and W3141A) (Fig. 4e). Notably, the 3D docking model of COL6A3-C5 and caMMP9 completely altered upon introducing these mutations, as predicted by AlphaFold, suggesting a substantail impact on their interaction (Fig. 4f). To validate this observation, we conducted co-IP assays with MMP9 and either C5 or C5^RFWmut^, revealing that point mutations within the C5 domain disrupted its interaction with MMP9 (Fig. 4g). Furthermore, ETP release from the C3C5 domain (pre-ETP) was markedly decreased by C3C5^RFWmut^ compared with that in the control (Fig. 4h). Notably, two specific amino acid sequences (307RSDY and 326FGF) located in the FN II repeat regions within the caMMP9 were predicted to be critical binding sites for the C5 domain (Fig. 4i**)**.

Meanwhile, to distinguish potential global structural perturbations from residue-specific functional effects, we generated alanine mutants (R3122A, F3124A and W3141A) and assessed their structural integrity and binding properties. As shown in Supplementary Fig. 2, structural alignment of the mutant and wild-type Col6a3-C5 models revealed no appreciable deviations in either secondary or tertiary structure. These results indicate that the mutations did not induce global conformational changes and therefore did not disrupt the overall structural framework of the C5 domain. Collectively, these findings suggest that the interaction between MMPs and the RFW residues (R3122, F3124 and W3141) within the C5 domain facilitates MMP-mediated cleavage at the C5 (L/M) site, thereby contributing to ETP generation, at least in part.

### NGC directly targets COL6A3-C5 to compete with various MMPs

**Fig. 5:**
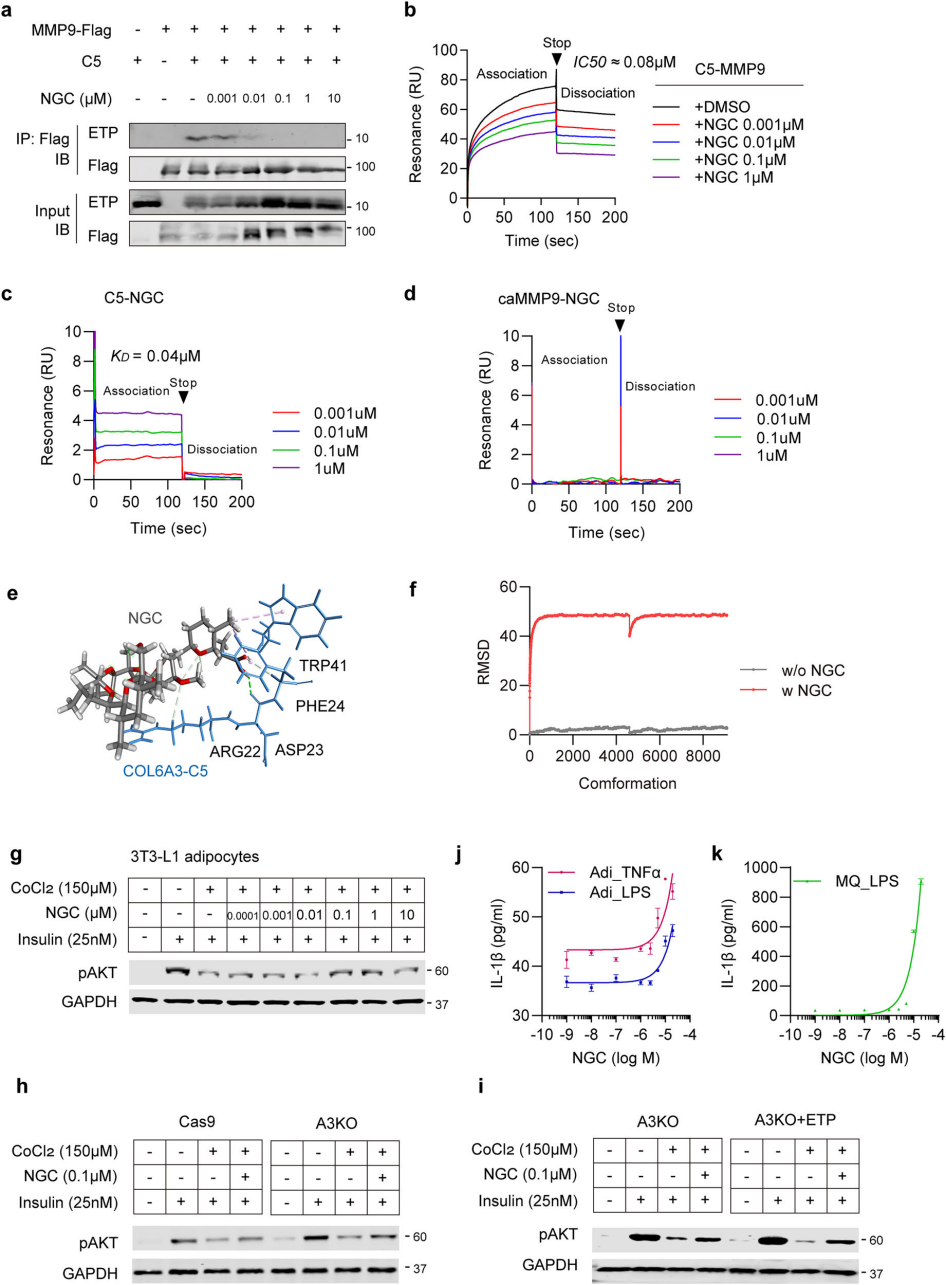
NGC disrupts the interaction between C5 domain of COL6A3 and MMPs. **a** Validation of the interaction between MMP9 and C5 domain of COL6A3 with various concentrations of NGC. HEK293T cells were transiently transfected with MMP9 and COL6A3-C5, and incubated with various concentrations of NGC. **b** Validation of COL6A3-C5 interaction with MMP9 with or without NGC by using SPR analysis. The recombinant COL6A3-C5 proteins were immobilized on the surface of the CM5 chip and then flowed over the MMP9 with NGC ranging from 0.001 to 1 µM. SPR binding curves for NGC and MMP9, binding to the captured COL6A3-C5 and fit to a 1:1 binding model. The half-maximal inhibitory concentration (IC_50_) value of NGC was approximately 0.08 µM. **c**, **d** Quantitative SPR measurements of immobilized either COL6A3-C5 (**c**) or MMP9 (**d**) interacting with NGC. **e** Molecular docking model illustrates the NGC and COL6A3-C5 binding interface. NGC adopts a stable binding pose within the COL6A3-C5 pocket (CDocker energy −8.5 kcal/mol, interaction energy −20.45 kcal/mol). The docking analysis identified R3122, D3123, F3124 and W3141 of COL6A3-C5 as key residues directly interacting with NGC. R3122 forms a carbon–hydrogen bond (2.98 Å) with the oxygen atom of the C-11 methoxy group of NGC, while D3123 establishes a conventional hydrogen bond (2.16 Å) with the hydroxyl group adjacent to the C-3 carbonyl. F3124 engages in both a carbon–hydrogen bond with the C-3 carbonyl oxygen (2.75 Å) and a π–alkyl interaction with the C-4 methyl group (4.40 Å). Similarly, W3141 forms a π–alkyl interaction (4.87 Å) with the C-4 methyl group. **f** NGC disrupts the structural stability of the MMP9–C5 complex. MD of the MMP9–C5 complex, performed with or without NGC, showed a stable conformation in the absence of NGC (RMSD 2.27 Å), whereas the presence of NGC markedly disrupted the binding interface (RMSD 47.99 Å). **g** Insulin responsiveness. The 3T3-L1 adipocytes were treated with CoCl_2_ (150 mM) for 48 h, followed by NGC for 16 h, before stimulation with insulin (25 nM) for 15 min. The levels of insulin-stimulated AKT phosphorylation were determined using immunoblotting. GAPDH was used as a loading control. **h**, **i** Insulin responsiveness of NGC in genetic depletion or supplementation of COL6A3/ETP. **h**, **i** A3KO (**h**) and ETP-reconstituted A3KO (**i**) adipocytes were treated with CoCl₂ (150 μM) for 48 h, followed by NGC for 16 h, and then stimulated with insulin (25 nM) for 15 min. Insulin-stimulated AKT phosphorylation was assessed by immunoblotting, with GAPDH serving as a loading control. The recombinant mouseETP (100ng/ml) was added to A3KO adipocytes to restore ETP levels. **j**, **k** The effect of NGC on inflammasome activation was assessed. The 3T3-L1 adipocytes (**j**) and peritoneal macrophages (**k**) were primed with LPS (1 µg/ml) or TNFα (50 ng/ml) for 6 h, followed by treatment with NGC. After 16 h, IL-1β concentrations in the culture supernatants were measured using enzyme-linked immunosorbent assay.

As MMP9 directly binds to COL6A3-C5 domain to proteolytically cleave, we investigated whether NGC alters MMPs’ recognition and binding affinity for the C5 domain. To check the optimal concentration of NGC required to alter the binding affinity of MMPs for COL6A3-C5 domain, we conducted co-IP assays using a range of NGC concentrations (1 nM to 1 µM) with caMMP9 and COL6A3-C5, showing that a low dose of NGC (1 nM) was sufficient to disrupt the interaction between COL6A3-C5 and caMMP9, with this effect being enhanced in a dose-dependent manner (Fig. 5a). Consistently, SPR analysis, one of the most sensitive assays for detecting direct molecular interactions, demonstrated a strong interaction between caMMP9 and COL6A3-C5, which was abrogated in a dose-dependent manner in the presence of NGC. Notably, NGC effectively disrupted this interaction at a low dose (1 nM) (Fig. 5b). To determine whether this inhibitory effect extends beyond MMP9, we performed IP analyses after cotransfecting FLAG-tagged MMPs (MMP1, MMP2, MMP9 and MMP16) with COL6A3-C5 in HEK293T cells, with or without NGC treatment. The results showed that NGC efficiently suppresses the interactions between COL6A3-C5 and all tested MMPs (Supplementary Fig. 3). Among them, the disruption of COL6A3-C5–MMP interactions was most pronounced with MMP9, although similar inhibitory trends were observed for the other MMPs as well. These findings indicate that NGC suppresses ETP generation, at least in part, by interfering with the interaction between COL6A3-C5 and multiple MMPs.

Next, we investigated whether NGC directly targets COL6A3-C5 or interacts with multiple MMPs simultaneously to alter their binding affinities. SPR analysis was conducted using either immobilized caMMP9 or COL6A3-C5 to assess their interactions with NGC. The results demonstrated that NGC specifically interacted with ETP, as indicated by a distinct binding signal when COL6A3-C5 was immobilized and NGC was applied (Fig. 5c). By contrast, when caMMP9 was immobilized, the addition of NGC produced no binding signal, indicating a lack of direct interaction (Fig. 5d). These findings suggest that NGC competes with MMP9 to target COL6A3-C5, thereby preventing the accessibility of MMPs to proteolytically cleave ETP.

Molecular docking analysis further supported this mechanism. NGC was predicted to stably occupy a defined pocket within the C5 domain (CDocker energy −8.5 kcal/mol) through hydrogen-bond and π-alkyl interactions with residues R3122, D3123, F3124 and W3141 (Fig. 5e**)**, consistent with the direct NGC–C5 domain interaction observed by SPR. To further validate these results, we conducted computational structural analyses integrating AlphaFold-based modeling, ZDOCK docking and atomistic MD simulations. In silico docking showed that the MMP9–C5 complex formed a stable interface in the absence of NGC (ZDOCK score 15.9), whereas inclusion of NGC markedly reduced the docking score (11.24), suggesting weakened binding. Likewise, MD simulations demonstrated that the MMP9–C5 complex remained structurally stable without NGC (average root mean square deviation (RMSD) 2.27 Å), but underwent substantial conformational disruption in the presence of NGC (average RMSD 47.99 Å) (Fig. 5f). Together, these computational findings indicate that NGC binds to the COL6A3-C5 domain to destabilize the MMP9–C5 complex and thus abolish their molecular interaction.

To determine whether the inhibitory effect of NGC on ETP generation contributes to enhanced insulin sensitivity in adipocytes, we used a model of hypoxia-induced insulin resistance in 3T3-L1 adipocytes. Treatment with NGC at concentrations ranging from 0.1 to 1 µM markedly improved insulin-stimulated AKT phosphorylation in CoCl_2_-induced hypoxic 3T3-L1 adipocytes (Fig. 5g).

COL6A3-KO adipocytes attenuated CoCl₂-induced suppression of insulin-stimulated AKT phosphorylation, supporting a role for COL6A3-derived ETP in this effect (Fig. 5h). Notably, NGC restored p-AKT levels even in COL6A3 KO adipocytes (Fig. 5h), indicating that its action is not dependent on ETP. Furthermore, ETP reconstitution in COL6A3-KO adipocytes had adverse effects on insulin signaling and enhanced insulin-stimulated Akt phosphorylation following NGC treatment (Fig. 5i). These data collectively suggest that the action of NGC is probably multifactorial, involving both inhibition of ETP generation and suppression of HIF-1α-dependent pathways.

NGC is well known as a potent inducer of the NLRP3 inflammasome^48^, leading to caspase-1 activation and the subsequent release of IL-1β, key processes in various inflammatory conditions^49,50^. Therefore, we investigated whether low concentrations of NGC, starting from 1 nM, which are sufficient to inhibit ETP generation, affect NLRP3-mediated inflammation in 3T3-L1 adipocytes treated with TNFα or lipopolysaccharide (LPS). The NLRP3-inflammasome pathway in adipocytes was activated by NGC at concentrations above 2.5 µM, as determined by the levels of IL-1β release in the conditioned media (Fig. 5j). Consistently, IL-1β release was also observed in LPS-stimulated macrophages treated with NGC at concentrations starting from 2.5 µM (Fig. 5k). Taken together, these findings highlight the dose-dependent effects of NGC; at lower concentrations, NGC is sufficient to compete with MMPs for ETP binding, whereas higher concentrations are required to trigger NLRP3-mediated inflammation responses in adipocytes.

### NGC administration ameliorates local fibroinflammation in AT and enhances systemic insulin sensitivity in HFD-induced obese mice

**Fig. 6:**
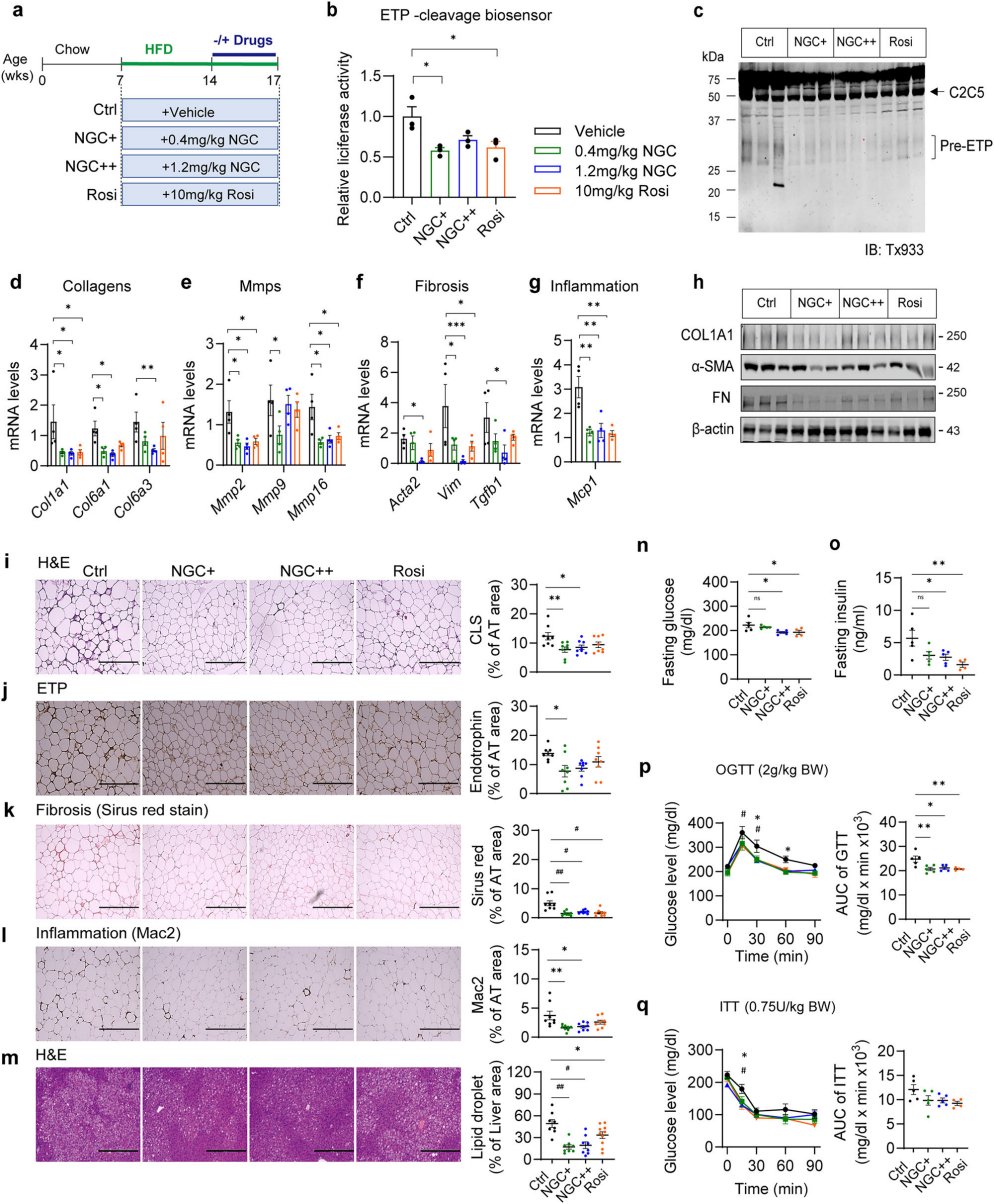
NGC alleviates AT fibroinflammation and improves insulin sensitivity in HFD-fed obese mice. **a** Experimental scheme of the administration of vehicle, NGC and rosiglitazone (Rosi) in HFD-induced obese mice. **b** Protease activities for ETP generation in the eWAT of HFD-fed obese mice treated with indicated drugs were determined using the ETP-cleavage biosensor overexpressing HEK293T cells. G-Luciferase activities were determined (*n* = 3/group). **c** Protease activity in eWAT for the generation of pre-ETP cleaved from C2C5 (*n* = 3 per group). The levels of cleaved pre-ETP from the C2C5 were assessed using immunoblotting with the anti-ETP antibody. **d**–**g** The mRNA levels of collagens (**d**), MMPs (**e**) and genes involved in fibrosis (**f**) and inflammation (**g**) in the eWAT of each group were determined using RT–qPCR (*n* = 4 per group). Statistical significance was evaluated using one-way ANOVA. *P < 0.05, **P < 0.01, ***P < 0.005. **h** The profibrotic protein levels (COL1A1, α-SMA and FN) in the eWAT were quantified using immunoblotting. β-Actin was used as a loading control. **i**–**m** Histological analysis: hematoxylin and eosin (H&E) staining (**i**), ETP (**j**), Sirius Red staining (**k**) and Mac2 (**l**) in eWAT from each group of mice, as well as H&E staining and quantification of the lipid droplets in the liver tissue of indicated mice (**m**). Scale bar, 400 µm. The proportion of positive-stained areas to total areas was estimated using ImageJ software. Statistical significance was evaluated using one-way ANOVA. *P < 0.05, **P < 0.01 (*n* = 8 per group). **n**–**q** Systemic insulin sensitivity. The level of glucose (**n**) and the insulin (**o**) measured in circulation following 4 h of fasting. Statistical significance was evaluated using two-way ANOVA. *P < 0.05, **P < 0.01. Insulin sensitivity was determined by OGTT (**p**) and i.p. ITT (**q**) after 2 weeks of drug treatment. Area under the curve (AUC) was calculated for each group, and statistical significance was evaluated using two-way ANOVA. *P < 0.05 versus *N* (1.2); #*P* < 0.05 versus Rosi (control (Ctrl), *n* = 5; NGC+, *n* = 5; NGC++, *n* = 5; Rosi, *n* = 4).

Next, we assessed the pharmacological potential of NGC in vivo by administering it to HFD-induced obese mice. Seven-week-old mice were fed an HFD for 7 weeks, after which body-weight-matched mice were separated into four groups and administered NGC (0.4 and 1.2 mg/kg body weight), rosiglitazone (Rosi, 10 mg/kg body weight) or vehicle (2% dimethyl sulfoxide/2% Tween-80 in PBS) via i.p. injection every other day for an additional 3 weeks of HFD feeding (Fig. 6a). Vehicle-injected mice were used as controls, while rosiglitazone, a well-known insulin sensitizer, was used as a positive control. NGC administration showed no significant differences in body weight or AT weight during the 3 weeks of treatment compared with controls, whereas slight weight gain was observed in the Rosi group, as expected (Supplementary Fig. 4a,b). Potential cytotoxicity of NGC in vivo was determined using the serum enzyme levels indicative of hepatic and renal toxicity, such as ALT, AST and BUN. No abnormal alterations were observed with NGC administration compared with those in the controls (Supplementary Fig. 4c–e).

To investigate the impacts of NGC on ETP generation in HFD-induced obese mice, we analyzed ETP cleavage activity in the AT extracts isolated from the epididymal white AT (eWAT) of each group of mice by using an ETP-cleavage biosensor, revealing that ETP cleavage activity was significantly decreased by NGC in obese mice (Fig. 6b). Consistently, the degree of C2C5 cleavage for pre-ETP generation was clearly decreased by NGC treatment, as determined by using immunoblotting (Fig. 6c). To examine whether NGC-mediated decrease of ETP levels contributes to local fibroinflammation in AT in obesity, marker gene expression for collagens, MMPs, fibrosis and inflammation in eWAT was analyzed. The gene expression for COL6 chains and MMPs associated with ETP levels was significantly decreased in NGC-treated obese mice as compared with that in the vehicle-treated control group (Fig. 6d,e). Along the same lines, marker genes for fibrosis and inflammation, including *Acta2*, *Vim*, *Tgfb1* and *Mcp-1*, were significantly decreased following NGC treatment compared with those in controls (Fig. 6f,g). At the protein level, fibrosis markers, such as COL1A1, α-SMA and fibronectin, were also consistently decreased in the eWAT of NGC-injected mice (Fig. 6h). Histological analysis further confirmed a significant reduction in crown-like structures in eWAT of NGC-treated obese mice, as indicated by H&E staining (Fig. 6i), in association with decreased levels of ETP (Fig. 6j). Increased levels of fibrosis and inflammation in obese eWAT were consistently decreased by NGC treatment, as determined using Sirius Red stains and Mac2 stains, confirmed a ~50% reduction in abnormal collagen deposition and macrophage infiltration, respectively (Fig. 6k,l). These results suggested that NGC protects against ETP generation and obesity-induced local fibroinflammation in AT. Notably, these effects were not associated with NGC-mediated activation of NLRP3 inflammasome responses (Supplementary Fig. 4f,g). Therefore, these results suggested that a low dose of NGC effectively ameliorates fibroinflammation in the eWAT of obese mice without activating NLRP3 inflammasome-mediated inflammatory responses.

To assess the impact of NGC on systemic glucose and insulin homeostasis, we conducted OGTTs and ITTs in HFD-fed obese mice. NGC treatment reduced fasting blood glucose levels by approximately 30% (Fig. 6n) and significantly lowered fasting insulin levels compared with those in controls (Fig. 6o). Glucose tolerance was markedly improved in NGC-treated mice, as indicated by a significant reduction in the area under the curve during OGTT (Fig. 6p). ITT results further demonstrated enhanced systemic insulin sensitivity following NGC treatment (Fig. 6q). In addition, NGC significantly alleviated hepatic steatosis (Fig. 6m) and modestly reduced circulating triglyceride and cholesterol levels in HFD-fed obese mice (Supplementary Fig. 4h,i). Collectively, these findings indicate that NGC improves systemic insulin sensitivity and attenuates hepatic steatosis in HFD-induced obese mice.

## Discussion

**Fig. 7:**
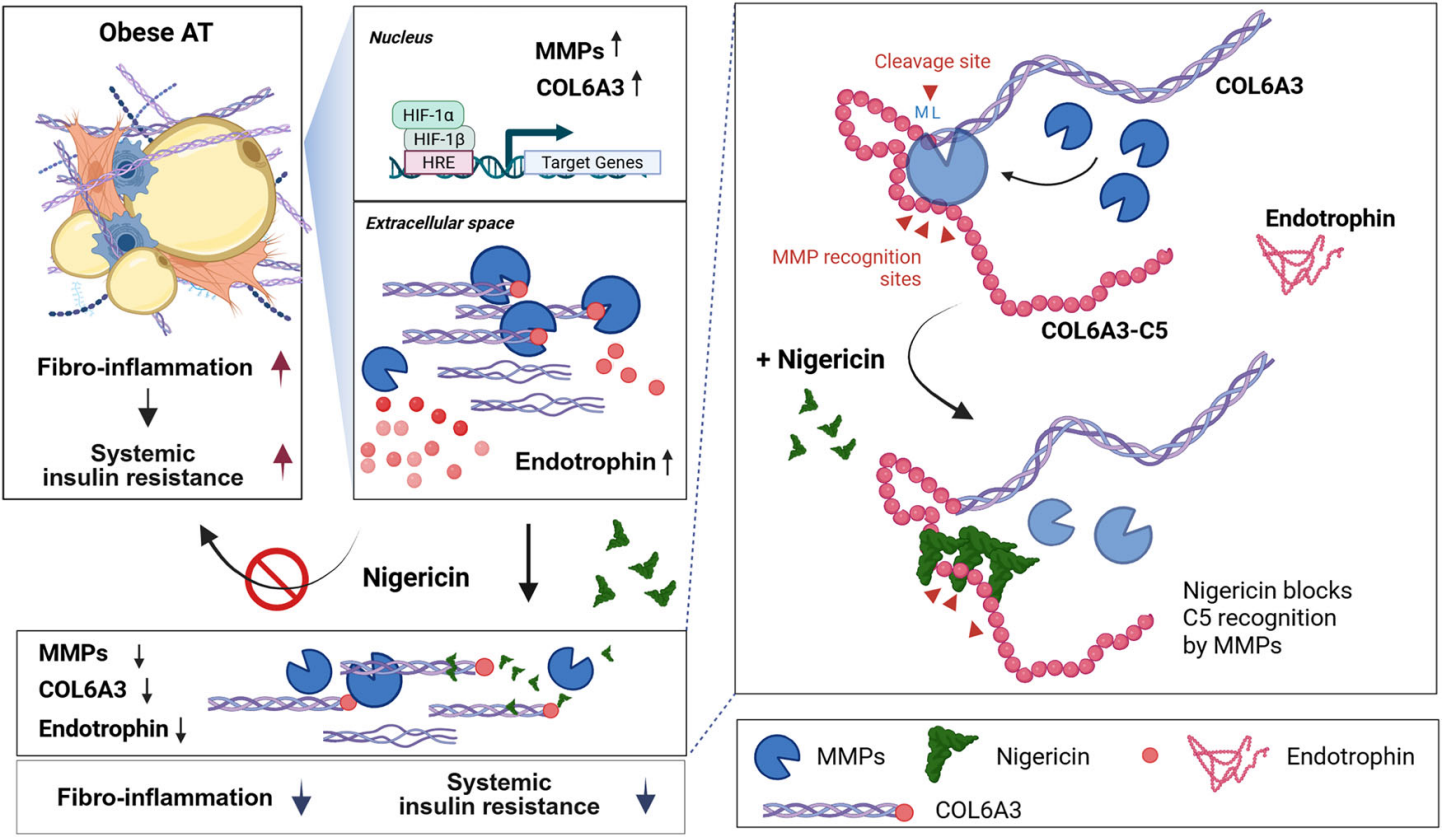
Schematic representation of the proposed mechanism by which NGC regulates ETP generation and improves insulin sensitivity in obese AT. In hypoxic obese AT, elevated HIF-1α induces gene expressions for the *COL6A3* and multiple *MMP*s involved in ETP generation, leading to ETP accumulation, fibroinflammation and systemic insulin resistance. Therapeutically, NGC directly binds to the COL6A3-C5 domain, competing with MMP biding sites, particularly MMP9, thereby preventing MMP recognition of COL6A3-C5 responsible for proteolytic cleavage of ETP. This inhibition decreases of ETP generation and alleviates fibroinflammation in AT and insulin sensitivity in obesity.

Recent advancements in drug development have focused on targeting proteins that regulate the microenvironment and their modifiers of obese AT to improve metabolic disorders^51^. Among these, HIF-1α is a promising therapeutic target. However, the development of clinically viable drugs remains a considerable challenge due to toxicity concerns that limit their suitability for clinical trials. Similarly, MMP inhibitors, which have shown potential in regulating ECM remodeling, are often associated with undesirable side effects, such as musculoskeletal pain and inflammation^52^. While these adverse effects are typically reversible, they necessitate dosage reductions that can compromise therapeutic efficacy. Given these limitations, there is a critical need for novel therapeutic interventions that can achieve maximum efficacy at low concentrations while minimizing toxicity and adverse effects. Of particular interest, ETP is a potent profibrogenic factor in obese adipose tissue, exacerbating metabolic diseases; therefore, ETP-inhibiting drugs could serve as promising therapeutic agents against obesity-related metabolic disorders. In this study, we demonstrate that NGC, a natural compound, significantly decreases the mRNA levels of *COL6A3* and *MMP*s, and inhibits the proteolytic cleavage of COL6A3 by MMPs to generate ETP. This effect is mediated by competing with the FNII domains of MMPs for interaction with the C5 domain of COL6A3, thereby preventing ETP cleavage from COL6A3. This inhibition was closely associated with a decrease in fibroinflammation in AT and systemic insulin resistance in both hypoxic adipocytes and diet-induced obese mice (Fig. 7). To the best our knowledge, NGC is the first natural compound to alleviate the levels of ETP generation.

The binding affinity and recognition of COL6A3-C5 by caMMP9 were effectively abolished at very low concentrations, ranging from 1 nM to 0.1 µM, and fibroinflammation and systemic insulin resistance in obese mice were effectively ameliorated at one-tenth of the dosage used for cancer treatment, without detectable cytotoxicity. Various studies have shown that NGC inhibits cancer cell growth, proliferation and migration at concentrations of 1–10 µM (refs. ^33,35^), while concentrations above 10 µM primarily induce cancer cell death. At relatively high concentrations, it induces apoptosis or necrosis by depleting intracellular potassium^53,54^, overcomes multidrug resistance and targets cancer stem cells^35,55,56^. In vivo, a dose of 4 mg/kg (i.p.) significantly delayed tumor growth and enhanced the efficacy of the chemotherapeutic agent, cisplatin, leading to reduced tumor volumes^33,57^. Thus, NGC is one of the drugs that exhibits a concentration-dependent therapeutic effect, indication and mode of action. For instance, rapamycin and sorafenib are also therapeutically used at different concentrations to different indications and modes of action^58–61^. Furthermore, the beneficial metabolic effects of low-concentration NGC in obesity observed in this study are independent of the NLRP3-mediated inflammasome pathway.

In general, substrate recognition and binding of MMPs to target substrates are critical steps for their digestion. MMP functions by directly binding to Ca^2+^ or Zn^2+^ ions at the active site^62^ and secondary protease binding sites (exosites), allosterically blocking the protease active site or preventing proMMP activation^63^. In particular, NSC405020 binds to the allosteric site of MMP2, altering the structure of enzyme and thereby hindering substrate binding, although it does not inhibit MMP2’s catalytic activity^64^. Similarly, NGC blocks the recognition of target substrate, particularly COL6A3, by multiple MMPs by directly targeting the COL6A3-C5 domain, rather than MMPs, which allows simultaneous inhibition of substrate recognition of multiple MMPs, without affecting MMP activity. Taken together, our findings revealed a previously unappreciated therapeutic effect and molecular mechanism of low-dose NGC treatment in obesity-induced metabolic diseases. The unique screening strategy used in this study contributes to the development of novel therapeutics to treat obesity-related metabolic disease, and application to patients with fibrotic disease and cancer who have pathologically high levels of ETP within the microenvironment.

## Supplementary information

Supplementary Information
