# Supplementary material for: Targeting COL6A3-C5 with nigericin suppresses endotrophin formation and enhances insulin sensitivity in obesity
Source: Exp Mol Med. 2026 Mar 5;58(3):768–81. doi: 10.1038/s12276-026-01661-y (PMC13049179; doi:10.1038/s12276-026-01661-y)
Supplement: Supplementary file 1 — Supplementary Information [file 12276_2026_1661_MOESM1_ESM.pdf]

Supplementary Information

Targeting COL6A3-C5 with nigericin suppresses endotrophin formation and enhances insulin sensitivity in obesity

Chu-Sook Kim[1#], Woobeen Jo[1#], Jungsun Yoo[1], Min Kim[1], JIn-Pyo An[2], Won-Keun Oh[2] and Jiyoung Park[1,2*]

[1]Department of Biological Sciences, [2]Graduate School of Health Science and Technology, College of Information and Biotechnology, Ulsan National Institute of Science and Technology, Ulsan 44919, Republic of Korea

[2]Research Institute of Pharmaceutical Sciences, College of Pharmacy, Seoul National University, Seoul 08826, Republic of Korea

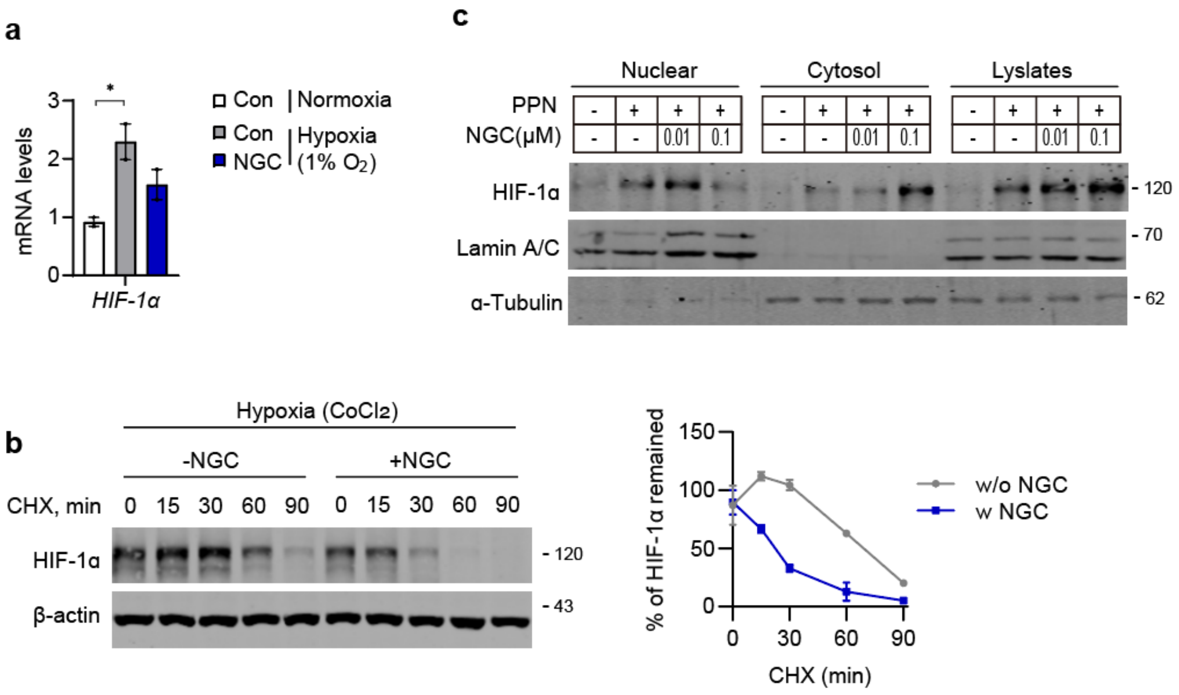

Supplementary Figure 1. NGC suppresses HIF-1α activity by reducing protein stability and inhibiting nuclear translocation. (a) RT-qPCR analysis of HIF-1α mRNA levels in 3T3-L1 adipocytes following treatment with NGC under hypoxia compared with those under normoxia for 24 h. Statistical significance was evaluated using one-way ANOVA. *$P<0.05$. (b) Cycloheximide (CHX) chase assay assessing HIF-1α protein stability. HEK293T cells were treated with $CoCl_2$ (400 μM, 4 h) to induce HIF-1α, followed by CHX (50 μg/ml) for the indicated times (0–90 min) in the presence or absence of NGC. Cell lysates were subjected to immunoblotting for HIF-1α and β-actin (loading control). The remaining HIF-1α protein level was quantified and presented as a percentage of the initial level. (c) Analysis of HIF-1α subcellular localization in HEK293T cells expressing HIF-1α-PPN following NGC treatment. Nuclear and cytosolic fractions were separated and immunoblotted for HIF-1α. Lamin A/C and α-Tubulin served as nuclear and cytosolic markers, respectively.

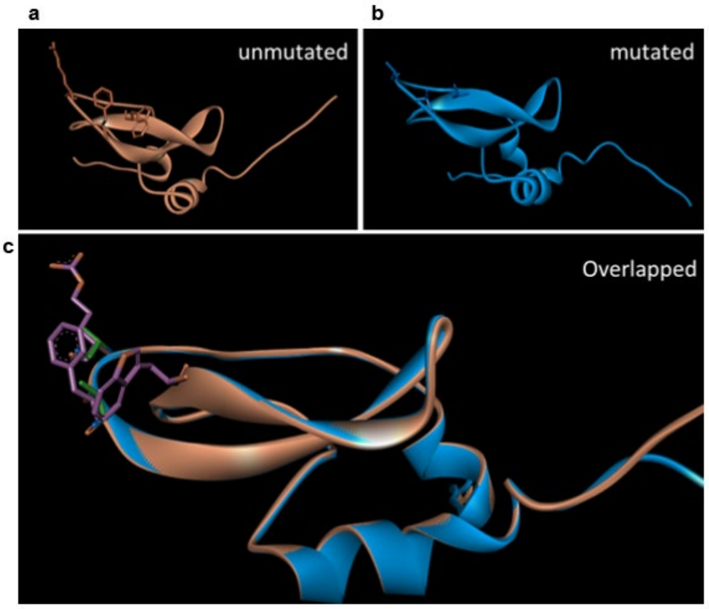

Supplementary Figure 2. Structural comparison of wild-type and alanine-substituted Col6a3-C5 variants. (a) Predicted 3D structure of the wild-type (unmutated) Col6a3-C5 domain. **(b)** Predicted structure of the alanine-substituted Mutated Col6a3-C5 (COL6A3-C5^{RFWmut}). **(c)** Structural overlap of wild-type (orange) and mutant (blue) Col6a3-C5 models. The two structures show nearly identical backbone conformations without detectable distortion, supporting that alanine substitutions do not induce global structural perturbation.

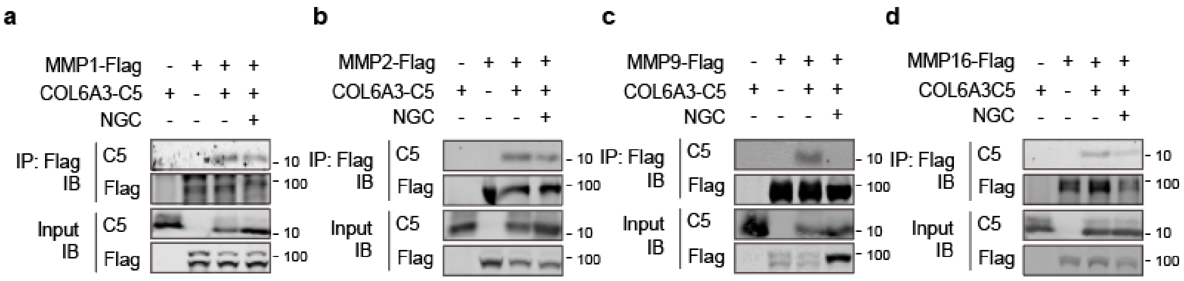

Supplementary Figure 3. NGC inhibits the interaction between the Col6a3-C5 domain and various MMPs. a–d HEK293T cells were transiently transfected with indicated MMP-FLAG (MMP-1, -2, -9, -16) and COL6A3-C5 constructs, following 24 h of incubation in the presence of 0.1 μM NGC. Cell lysates were immunoblotted using anti-FLAG and anti-C5 antibodies. Immunoblots for indicated MMPs and C5 expression followed by immunoprecipitation (IP) of MMP-FLAG by FLAG conjugated beads.

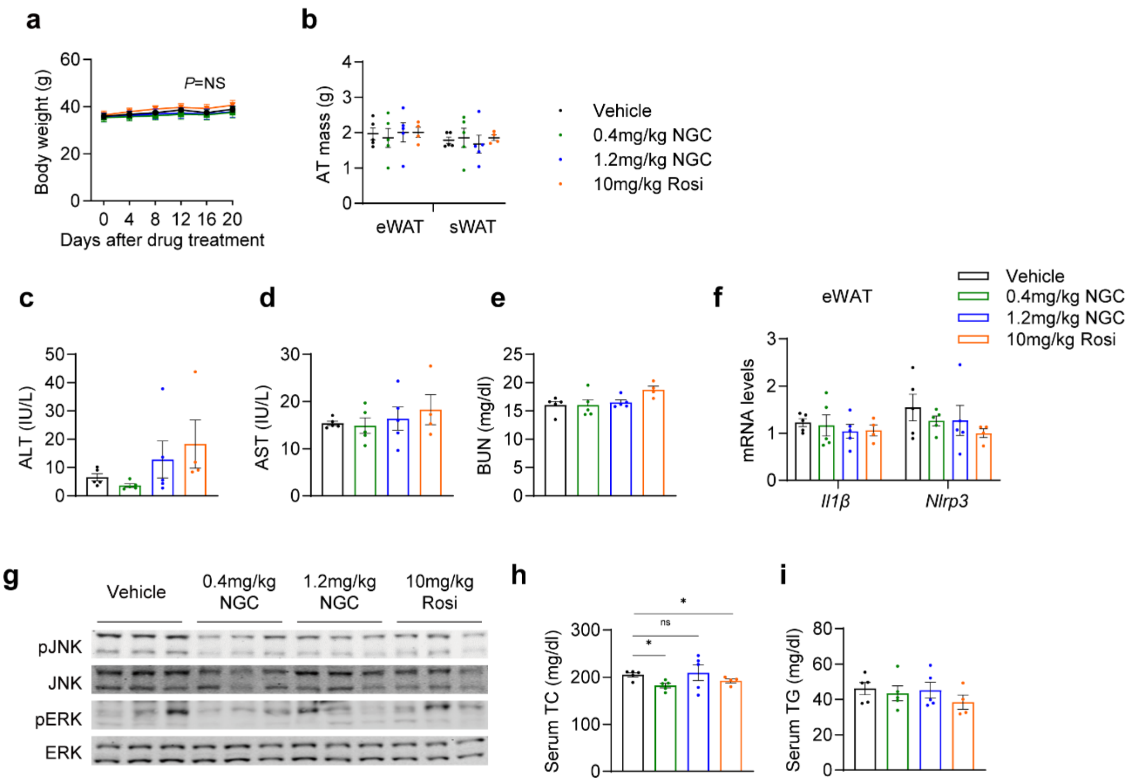

Supplementary Figure 4. The metabolic effects of NGC in HFD-induced obese mice. Administration of vehicle, nigericin (NGC), and rosiglitazone (Rosi) for 3weeks in HFD-induced obese mice (n=4-5 for each group). Body weight changes (**a**) and AT mass (**b**). (**c-e**) Systemic cytotoxicity assessments in obese mice were measured by enzymatic assay**.** Serum levels of ALT (**c**), AST (**d**) and BUN (**e**). **(f-g)** Inflammatory phenotypes in eWAT of each group of mice were determined by RT-qPCR and western blots (n=4-5 per group). (**f**) The mRNA levels of Il1b and Nlrp3, (**g**) The phosphorylation of ERK and JNK. **(h, i)** Lipid profiles in serum in each group of obese mice. The levels of Total cholesterol (**h**) and Triglyceride (**i**). Statistical significance was evaluated by one-way ANOVA. *P<0.05.

Supplementary Table 1. List of antibodies used in this study

Name	Company	Catalog	Species
Mouse ETP	COVANCE	Tx621	Rabbit
Human ETP	COVANCE	Tx933	Rabbit
HIF1α	CST	D1S7W	Rabbit
Anti-FLAG	Santa Cruz	sc-166384	Mouse
Anti-V5	Santa Cruz	sc-81594	Mouse
β-actin	Santa Cruz	sc-47778	Mouse
GAPDH	Santa Cruz	sc-47724	Mouse
MAC2	Santa Cruz	sc-32790	Mouse
COL1A1	Santa Cruz	sc-293182	Mouse
Fibronectin	Santa Cruz	sc-271098	Mouse
Anti-alpha smooth muscle Actin	Abcam	ab5694	Rabbit
SAPK/JNK	CST	9252s	Rabbit
P-SAPK/JNK (T183/Y185)	CST	4668s	Rabbit
p44/42 MAP Kinase	CST	4695s	Rabbit
P-p44/42 MAPK (T202/Y204)	CST	9106s	Mouse
Akt	CST	9272S	Rabbit
P-Akt (S473)	CST	9271S	Rabbit

Supplementary Table 2. List of chemicals used in this study

Name	Company	Catalog
Nigericin	Enzo Life Sciences	BML-CA421-0005
G-fectin transfection reagent	Genolution	
Cobalt(II) chloride	SigmaAldrich	232696
Coelenterazine	SigmaAldrich	C2230
Adenosine 5′-triphosphate disodium salt hydrate	SigmaAldrich	A2383

Supplementary Table 3. List of primer sequences used for qRT-PCR analysis

Genes	Species	Primer sequences
Col6a3	Mouse	F-5'- CAGAACCATTGTTTCTCACT -3'<br>R-5'- AGGACTACACATCTTTTCAC -3'
Col1a1	Mouse	F-5'- CCTCAGGGTATTGCTGGACAAC -3'<br>R-5'- CAGAAGGACCTTGTTTGCCAGG -3'
Col3a1	Mouse	F-5’- GACCAAAAGGTGATGCTGGACAG -3’<br>R-5’- CAAGACCTCGTGCTCCAGTTAG -3’
Col6a1	Mouse	F-5’- GCGCTTCATTGACAACCTGAGA -3’<br>R-5’- CTCCTCCAGCCCCTTCTT -3’
18s	Mouse	F-5'- AGGGTTCGATTCCGGAGAGG -3'<br>R-5'- CAACTTTAATATACGCTATTGG -3'
Hif1a	Mouse	F-5'- GATTCGCCATGGAGGGC -3'<br>R-5'- AGACTCTTTGCTTCGCCGAG -3'
Glut1	Mouse	F-5'- GGTGTGCAGCAGCCTGTGTA -3'<br>R-5'- CAACAAACAGCGACACCACAGT -3'
Pdk1	Mouse	F-5'- GCAGCAGAGAGTAAACTGTTTG -3'<br>R-5'- TGGTCACCTGACCTCTCG -3'
Tgfb1	Mouse	F-5'- GACCCTGCCCCTATATTTGGA -3'<br>R-5'- CCGGGTTGTGTTGGTTGTAGA -3'
Fn	Mouse	F-5'- ATGTGGACCCCTCCTGATAGT -3'<br>R-5'- GCCCAGTGATTTCAGCAAAGG -3'
Vim	Mouse	F-5'- GCCAGCAGTATGAAAGCGTG -3'<br>R-5'- ACCTGTCTCCGGTACTCGTT -3'
Acta2	Mouse	F-5'- GTCCCAGACATCAGGGAGTAA -3'<br>R-5'- TCGGATACTTCAGCGTCAGGA -3'
Timp1	Mouse	F-5’- TACACCCCAGTCATGGAAAGC -3’<br>R-5’- CGGCCCGTGATGAGAAACT -3’
Mmp1	Mouse	F-5’- AACTACATTTAGGGGAGAGGTGT -3’<br>R-5’- GCAGCGTCAAGTTTAACTGGAA -3’
Mmp2	Mouse	F-5'- CAAGTTCCCCGGCGATGTC -3'<br>R-5'- TTCTGGTCAAGGTCACCTGTC -3'
Mmp9	Mouse	F-5'- CTGGACAGCCAGACACTAAAG -3'<br>R-5'- CTCGCGGCAAGTCTTCAGAG -3'
Mmp16	Mouse	F-5'- CTGACAAGATCCCTCCACCTAC -3'<br>R-5'- GTGTTGAAGTTCCCATCACAGA -3'
Mcp1	Mouse	F-5'- CTGGATCGGAACCAAATGAG -3'<br>R-5'- CGGGTCAACTTCACATTCAA -3'
Il6	Mouse	F-5'- TCGTGGAAATGAGAAAAGAGTTG -3'<br>R-5'- AGTGCATCATCGTTGTTCATACA -3'
Tnfa	Mouse	F-5'- CATCTTCTCAAAATTCGAGTGACAA -3'<br>R-5'- TGGGAGTAGACAAGGTACAACCC -3'
Il1b	Mouse	F-5'- GCAACTGTTCCTGAACTCAACT -3'<br>R-5'- ATCTTTTGGGGTCCGTCAACT -3'
Nlrp3	Mouse	F-5'- TCACAACTCGCCCAAGGAGGAA -3'<br>R-5'- AAGAGACCACGGCAGAAGCTAG -3'

F: Forward primer, R: Reverse primer
